# New strategy for testing efficacy of immunotherapeutic compounds for diabetes in vitro

**DOI:** 10.1186/s12896-016-0270-0

**Published:** 2016-05-10

**Authors:** Gecilmara Salviato Pileggi, Aline Dayana Clemencio, Thiago Malardo, Sonir R Antonini, Vania Luiza Deperon Bonato, Wendy Martin Rios, Celio L Silva

**Affiliations:** Department of Pediatrics, Ribeirão Preto Medical School, University of São Paulo (USP), Av. Bandeirantes, 3900, 7 Floor, 14049-900 Ribeirão Preto, SP Brazil; Department of Biochemistry and Immunology, Ribeirão Preto Medical School, USP, Ribeirão Preto, SP Brazil

**Keywords:** Diabetes, Heat-shock proteins, Regulatory T cells, Th17 cells, Immunotherapy, NOD mice

## Abstract

**Background:**

The valuable role of immunotherapy in treating autoimmune diseases is increasingly recognized by those involved in the research and clinical application of new biopharmaceuticals products. However, many aspects related to the mechanisms of immune-modulated therapies remain to be elucidated in order to explore fully the emerging opportunities. The non-obese diabetic NOD mouse develops insulin-dependent diabetes mellitus spontaneously as a consequence of an autoimmune process in the presence of pathogenic CD4^+^ T cells that typically exhibit Th17 cell phenotypes. The change of a Th17 phenotype into a pattern of regulatory T cells (Treg) is extremely important in controlling autoimmune diseases. Heat shock proteins (HSPs) are stress-induced proteins with immunoregulatory properties. In the current study, the capacity of Hsp65 and Hsp70 mycobacterial HSPs and a constructed DNA encoded Hsp65 (DNAhsp65) to transform the pattern of the immune response from Th17 into Treg cells has been studied in vitro using co-cultures of antigen presenting cells (APCs) and T cells in NOD mice.

**Results:**

Cells harvested from NOD mice and cultured for 48 h (without immunoregulatory compounds) presented with Th1/Th17 patterns and secretions of IL-6, IFN-*γ*, IL-10 and IL-17 cytokines. The cultured cells from the non-diabetic BALB/C mice exhibited a Th1 pattern and the production of IL 6 and IFN-γ secretions. An up-regulation was observed in the supernatants from the co-cultures of NOD cells that were stimulated with DNAhsp65, Hsp65 or Hsp70 through increased levels of IL-10 secretion and the suppression of IL-6, IFN-γ and IL-17 production. In addition, immunoregulation was demonstrated through IL-17 suppression in the co-culture stimulated by the specific insulin antigen. Moreover, an increase of immunoregulatory compounds were observed in the co-culture through the expression of CD11b^+^CD86^+^ activation markers on APCs, as well as the frequency of Treg cells expressing CD4^+^CD3^+^ and CD4^+^CD25^hi^.

**Conclusions:**

The in vitro observation of Th17 cells differentiating into Tregs in NOD mice could raise the hypothesis that the immune regulatory activity of HSPs could be an efficient strategy for diabetes prevention and treatment.

## Background

Diabetes mellitus develops spontaneously in NOD mice and because of the range of tools and genetic information available for mouse studies, the NOD mouse has become the model of choice for laboratory studies. Studies using NOD mice have provided a basic understanding of disease mechanisms and immunological tolerance that have enabled observation and gathering of information about the functioning of pathogenic T cells that would otherwise not been possible without this model. **Insulin-dependent** diabetes mellitus is characterized by increased T cell response toward several auto antigens, including Hsp60, glutamic acid decarboxylase and insulin [1, 2]. The primary mediators of β-cell destruction are CD4^+^ and CD8^+^ T cells [3, 4]. Pathogenic CD4^+^ T cells typically exhibit Th1 and Th17 cell phenotypes, characterized by increased secretion of several mediators of the immune response including IFN-γ, TNF-α, IL-6, IL-10 and IL-17 [5]. Moreover, regulatory T cells (Tregs) play an important role in the control of autoimmune illnesses, especially in diabetes [6]. Using NOD mice as an experimental model of diabetes therefore is instrumental in the pursuit of potential innovative immunotherapies to treat and resolve insulin-dependent diabetes mellitus.

Heat shock proteins (HSPs) are stress-induced proteins with immunoregulatory properties [7–10]. Immune responses to HSPs develop in virtually all inflammatory diseases and the mycobacterial HSPs, mainly Hsp65 and Hsp70, are known to modulate both the innate and adaptive host aspects of the immune system [7, 8]. The mycobacterial Hsp65 as a DNA vaccine construct (DNAhsp65) has also been the focus of tolerogenic immune interventions aimed at preventing and treating-mediated diseases such as experimental autoimmune encephalitis, arthritis, atherosclerosis and diabetes [11–14]. Other immunotherapeutic or immunoregulatory effects of DNAhsp65 were also described by our group for infectious diseases [15], cancer [16], and allergy [17].

We have also shown recently that a DNAhsp65 construct is protective against experimental diabetes in NOD mice [14]. The protective effect was associated with a clear shift in the cellular infiltration pattern in the pancreas. This change included reduction of CD4^+^ and CD8^+^ T cells infiltration, appearance of CD25^+^ cells influx and an increased staining for interleukin IL-10 in the islets [14].

In this study, we investigated the ability of Hsp65, Hsp70 and DNAhsp65 to modify the pattern of immune response from one that is Th17 to one of Treg cells in a co-culture of APCs and T cells of NOD mice. We compared the effect of immunoregulatory compounds on the changes in the production of Th1 and Th17 cytokines by co-cultured cells. Moreover, we analyzed the changes in the frequency of CD4^+^ T cells, especially the CD4^+^CD25^hi^ Tregs, and the expression of CD11b^+^CD86^+^ activation markers on APCs in co-cultures. Our data suggest there are an influence of HSPs on the up-regulation of Tregs in NOD cells based on the changes in the production of cytokines and the expression of activation markers in a co-culture of T cells and antigen presenting cells (APCs).

## Methods

### Animals

NOD/Uni mice used in this study derived from the INSERM U-25 colony at the Hospital Necker (Paris, France) and had been maintained under germ-free conditions at the animal breeding centre of the State University of Campinas, São Paulo, Brazil. Twenty female NOD mice were used in the experiments and insulitis begins around 4 weeks of age and overt hyperglycemia was observed clearly at around 16 weeks when they were sacrificed. Female, Specific Pathogen-Free, BALB/c mice (6–8 weeks old) from the Animal Facility of the Medicine School of Ribeirão Preto, University of São Paulo, was also used in the experiments.

The animals were maintained on a 12-h light/dark cycle and given free access to food and autoclaved water. The experimental protocol was conducted according to the Ethical Principles In Animal Research adopted by the Brazilian College of Animal Experimentation (COBEA) and was approved by the Ribeirão Preto School of Medicine of University of São Paulo – Ethical Commission of Ethics in Animal Research (CETEA) (protocol 042/2011).

### Achievement of peritoneal macrophages

Mice were euthanized with an overdose of anesthetic, ketamine hydrochloride (50 mg/kg) and xylazine (15 mg/kg). To harvest peritoneal cells, 5 mL of phosphate buffered saline were injected (PBS 10 mM, pH: 7.2 at 4 °C) in the peritoneal cavity. Thereafter, the cell solution was aspirated and transferred to a Falcon tube and centrifuged at 450 × g for 10 min at 4 °C. After centrifugation, the cell pellet was resuspended in fresh RPMI medium. The cells were characterized by FACScan using the following monoclonal antibodies: CD11b-PeCY5 (clone M1/70), CD40-APC (clone 3/23), CD86-PE (cloneGL). The percentage of macrophages recovered from the peritoneal cavity was 58 %. The number of viable cell concentration was adjusted to 2 × 10^5^cells/mL.

### Achievement of splenic cells

After sacrifice and paracentesis, the animals splenectomy was performed and spleens cells were recovered and washed twice with RPMI 1640 medium (GIBCO) without added serum and supplemented with 2 mM L-glutamine (Gibco), 1 % 100 mM sodium pyruvate (Gibco), 2 % non-essential amino acid solution 50 × (GIBCO), 1 % 100× solution of vitamins (GIBCO) and 0.1 % gentamicin (Novafarma). For these washes, the cells were centrifuged for 8 min at 800 g and 400 g, respectively.

Finally, the cell pellet was resuspended in 10 mL complete RPMI 1640 medium consisting of 90 % RPMI 1640 medium supplemented as described above and 10 % Fetal Bovine Serum (FBS) (Hyclone). Then the counting of spleen cells was done in a Neubauer chamber. Differential cell count was promoted in a hematology analyzer ABX Micros 60® (HORIBA ABX Diagnostics Inc., Kyoto, Japan).

### Separation of CD4^+^ T lymphocytes

CD4+ and CD8+ T lymphocytes were separated by negative selection using Magnetic Beads (Stemcell Technologies Inc., Vancouver, Canada) according to the manufacturer’s instructions. The viability and cell concentration was performed counting in a Neubauer chamber with Trypan blue dye. An aliquot of CD3+ cells recovered after separation with magnetic beads was evaluated by flow cytometry to determine the degree of purification obtained in the separation process. The cells analyzed by flow cytometry were incubated with specific monoclonal antibodies (PharMingen, San Diego, USA) for 30 min at 4 °C. The cells were characterized by FACScan using the following monoclonal antibodies were used: CD3–FITC (clone MEL-14), CD4-APC (clone IM7), CD8–Percp (clone 53–6.7), CD25-PE (clone RM4-5). After the incubation period, the samples were centrifuged at 400 × g for 10 min at 4 °C. The supernatants poured and samples were washed with 2 mL FACS buffer (PBS plus 2 % FCS) and centrifuged again at 400 × g for 10 min at 4 °C. At the end of the process, the cells were fixed with 200 of PBS plus 1 % formaldehyde (JT Baker, Xalostoc, Mexico). The acquisition of samples was performed in FACSort (Becton & Dickinson) and the analysis in Cell Quest program from 25,000 acquired events. The cell population obtained was selected through size parameters (FSC) and granularity (SSC) and within the selected population, the percentage of cells by double staining was evaluated.

### Co-culture of peritoneal macrophages and CD4^+^ T lymphocytes

Peritoneal macrophages were distributed into 48 well culture plates, about 2 × 10^5^ per well in flat bottom plates (Corning, NY, USA), and resuspended in RPMI medium supplemented with 10 % fetal bovine serum, 100 U/mL penicillin, 100 ug/mL streptomycin, 100 μg/mL gentamicin, 2 mM L-glutamine, 50 mM mercaptoethanol, 1 mM sodium pyruvate. The plates were incubated at 37 °C with an atmosphere of 5 % CO2. After 2 h incubation, the nonadherent cells were removed gently with cold PBS aid. Approximately 10 × 10^5^ CD4+ T lymphocytes in 500 μL of complete RPMI medium were added do the cultures.

### Co-culture stimulation

In the plates containing macrophages and CD4^+^ T lymphocytes from diabetic NOD or control BALB/c mice were co-cultured separately in the presence of DNAhsp65 (50 μg), Hsp65 (5 μg), or Hsp70 (5 μg). As a negative control, cells were co-cultured only in the presence of RPMI complete culture medium. As a positive control, cells were stimulated with Concavalin A (ConA, 40 μg) or insulin (20 μg/mL).

### DNAhsp65 and recombinant Hsp65 and Hsp70 proteins purification

DNA plasmid expressing Hsp65 (DNAhsp65) and the recombinant mycobacterial Hsp65 and Hsp70 were obtained as previously described [16, 17]. Protein characterization was performed by electrophoresis on acrylamide gel developed with 10 % Coomassie Brilliant Blue R 250 (Ultra Pure). In parallel, it was determined the protein concentration by the Bradford method (Comassie® Protein Assay Reagent, Pierce Chemical).

### Endotoxin determination

The endotoxin concentrations in samples of recombinant protein and plasmid DNA were determined by LAL technique using the LAL QCL −1000 kit (Cambrex Company, Walkersville, MD, USA). The content of endotoxin in the samples was 0.09 EU endotoxin units/μg protein. According to FDA specifications, samples containing values between 0.05 and 0.1 EU endotoxin units/μg protein are considered appropriate.

### Glucose determination

Mice were monitored for the onset of diabetes by measurements of blood glucose using Prestige LX Smart System test-strips (Home Diagnostic, Inc., Fort Lauderdale, FL, USA). Consecutive readings of blood glucose levels = 200 mg/dL (12 mmol/L) accompanied by glycosuria on 2 consecutive days were considered to be diagnostic of diabetes onset [13]. The incidence is expressed as percentages.

### Cytokine detection

After the culture period of macrophages and lymphocytes for 48 h, with or without stimulus, the culture plates were centrifuged at 800 g for 10 min. The supernatants were collected and stored in a freezer at −70 °C for later titration of cytokines. The determination of the concentration of IL-6, IL-10, IL-17, and IFN-γ in the supernatant of the co-cultures was performed by using a commercial immunoassay kit Milliplex Magpix® Rat/Mouse (EMD Millipore Co., Billerica, MA, USA) for Luminex™ x MAP technology. The fluorescence intensity was measured and the mean cytokine concentrations were calculated as pg/mL.

### Statistical analysis

All the experimental data are expressed as means ± SEM and the statistical analyses was performed using the GraphPadPrism software, version 6.0 (GraphPad Software, San Diego, USA). The results were obtained from three independent experiments. The Student’s *t*-test was applied for two-group comparison or one-way ANOVA with Dunnett’s post-test (medium as reference group) for multiple comparison. Values of P < 0.05 were considered statistically significant.

## Results

In this work we observed that insulitis in NOD mice begins around 4 weeks of age and overt hyperglycemia was detected clearly at around 18 weeks and cumulative insulin-dependent diabetes mellitus incidence reaches 50 % or even more at 22–23 weeks (data not shown). To evaluate the inflammatory/immune conditions of NOD mice in comparison with normal non-diabetic BALB/c mice we first determined the cytokines released in the supernatants of APCs and CD4^+^ T cells co-culture. As shown in Table 1, high levels of IL-6, IL-10, IL-17, and IFN-γ cytokines were detected in the supernatant of co-cultures from NOD mice when compared to cells from BALB/c mice. Contrary to the findings for the cytokines of NOD mice, the levels of IL-17 and IL-10 were low in BALB/c mice even under Con-A stimulation. The high levels of cytokines observed in NOD animal cells without stimulation (media control) may be due to the previous state of activation of the immune system of these animals with diabetes when compared with the data obtained from BALB/c mice. These findings show that antigen specific immune response in diabetic mice is most commonly associated with Th1/Th17 pattern of immune response, although insulin also stimulates IL-10 production.

**Table 1:** Analysis of cytokine released (pg/mL) in the supernatant of co-culture of APCs and T lymphocytes from NOD and BALB/c mice stimulated with medium, Con-A, or insulin

Cytokine	Medium		Con-A		Insulin	
	NOD	BALB/C	NOD	BALB/C	NOD	BALB/C
IL-6	6234 ± 3217^a^	494 ± 377	9660 ± 2001	7632 ± 2104	13,881 ± 1138^b^	2762 ± 261
IFN-Υ	96 ± 8^a^	4.5 ± 0.1	4754 ± 1725	3253 ± 3224	480 ± 204^a^	13 ± 11
IL-17	368 ± 27^b^	23.6 ± 22	680 ± 219^a^	28.8 ± 20.7	984 ± 34^a^	53 ± 26
IL-10	89 ± 12^a^	1.8 ± 0.5	758 ± 137^b^	50 ± 35	595 ± 118^b^	33 ± 21

Data are reported as means ± SD for 3 separate experiments

^a^ *P* < 0.001

^b^ *P* < 0.05 when compared BALB/c to NOD mice (*t*-test)

**Fig. 1:**
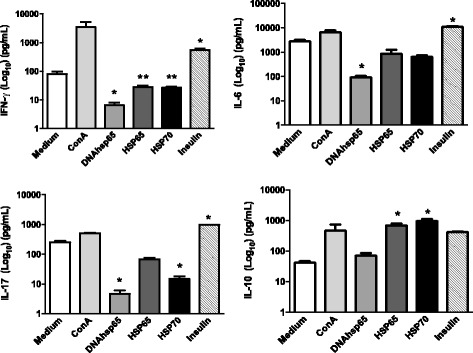
Effect of DNAhsp65, Hsp65 or Hsp70 on IL-6, IFN-γ, IL-10 and IL-17 secretion in co-culture of APCs and CD4^+^ T cells from NOD mice for 48 h. The levels of cytokines were determined using MilliplexMagpix assay. Data are reported as means ± SD for 3 separate experiments. *P < 0.001; ** *P* < 0.05 compared to insulin control (*t*-test)

DNAhsp65 and the recombinant Hsp65 and Hsp70 proteins modulate the levels of Th1 and Th17 cytokines in co-cultures of NOD cells. Indeed, as shown in Fig. 1, all the compounds down modulated the production of IL-6 (*P* < 0.05 for DNAhsp65 and not significantly different for Hsp65 and Hsp70), IL-17 (*P* < 0.001 for DNAhsp65 and Hsp70 and not significantly different for Hsp65), and IFN-γ (*P* < 0.001 for DNAhsp65 and *P* < 0.05 for Hsp65 and Hsp70). The various treatment conditions also stimulated positively the production of IL-10 (*P* < 0.05 for Hsp65 and Hsp70 and not significantly different for DNAhsp65). Thus, the suppression of Th1 and Th17 cytokines were clearly detected in the presence of these compounds indicating that they exhibited clear immune regulatory activity in co-cultures.

The addition of immunoregulatory compounds in the co-cultures promotes the activation of APCs and generation of CD4^+^CD25^hi^ cells, a hallmark or regulatory T cells (Treg). Treatment with DNAhsp65, Hsp65 or Hsp70, in the presence of insulin, caused a significant increase to the expression of the activation marker CD11b^+^CD86^+^ by APCs when compared to those untreated (Table 2). As for T cells, a significant increase in the frequency of CD4^+^CD25^hi^ cells, passing from 1.2 ± 0.6 without immunoregulatory to 6.7 ± 1.6, 9.6 ± 3.7 and 7.7 ± 1.7 in the presence of DNAhsp65, Hsp65, and Hsp70, respectively (*P* < 0.001). As shown in Table 2, the same pattern was observed for the frequency of cells expressing CD4^+^CD3^+^ under stimulation with insulin plus DNAhsp65, Hsp65 or Hsp70 (*P* < 0.05 compared with the insulin group).

**Table 2:** Analysis of T CD4 lymphocyte expression of CD4^+^CD3^+^ and CD4^+^CD25^hi^, and CD86 activation markers in APCs

Treatment	Percent of cell phenotypes		
	CD4^+^CD3^+^	CD4^+^CD25^hi^	CD11b^+^CD86^+^
Medium	24.5 ± 8.2	0.5 ± 0.2	31.8 ± 7.1
Insulin	34.4 ± 3.8	1.2 ± 0.6	44.8 ± 8.6
Insulin + DNAhsp65	49.2 ± 6.8^a^	6.7 ± 1.6^b^	77.6 ± 5.1^b^
Insulin + Hsp65	42.8 ± 4.5^a^	9.6 ± 3.7^b^	69.3 ± 6.0^b^
Insulin + Hsp70	43.1 ± 3.9^a^	7.7 ± 1.7^b^	65.0 ± 7.4^b^

Data are reported as means ± SD for 3 separate experiments

^a^ *P* < 0.05 compared to insulin group (*t*-test)

^b^ *P* < 0.001

**Fig. 2:**
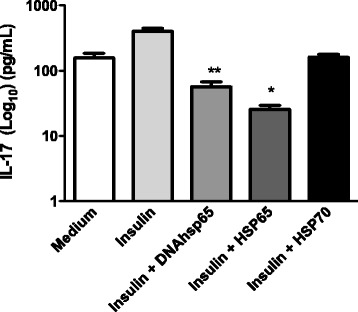
Effect of DNAhsp65, Hsp65 or Hsp70 on IL-17 secretion in co-culture of APCs and CD4^+^ T cells from NOD mice for 48 h in the presence of insulin. The levels of cytokines were determined using MilliplexMagpix assay. Data are reported as means ± SD for 3 separate experiments. *P < 0.001; ** *P* < 0.05 compared to insulin control (*t*-test)

To further confirm the possible role of HSPs in immune regulation of Th17 cytokine secretion in co-culture, IL-17 was examined properly. As shown in Fig. 2, we found that the various treatment conditions suppressed IL-17 secretion (*P* < 0.001 for Hsp65, *P* < 0.05 for DNAhsp65 and not significantly different for Hsp70) in the supernatants of co-cultures stimulated with the specific antigen insulin. These data suggest that immunomoregulatory compounds might reduce the Th17 cell population resulting in suppression of IL-17 cytokine production. Moreover, concomitant with suppression in Th17, immunoregulatory induced generation of Treg cells that are important in the control of diabetes with a concomitant increased level of IL-10 production and decreased in IL-6 and IFN-γ.

## Discussion

Immunotherapy has made a significant therapeutic impact in immunologically mediated diseases such as cancer and allergy, but the search for safe and effective immunotherapy continues in autoimmune diseases such as arthritis and diabetes [18, 19]. It has been estimated that screening experiments of new immunotherapeutic compounds would require appropriate experimental model, long time to test and use of large number of animals. Therefore, it is very important to develop some strategies for predicting immune regulatory effects of new compounds in-vitro. NOD mice acquired diabetes with a characteristic inflammatory Th1 and Th17 pattern of immune response [20–22].

Our study has shown for the first time that T lymphocytes obtained from NOD animals exhibit in vitro a pattern profile of Th1 and Th17 when compared to cells obtained from BALB/c. In particular, IL-6, IFN-gamma, IL-10 and IL-17 were secreted in the supernatants of co-cultures of CD4^+^ T cells and APCs from NOD mice. This kind of immune response is consistent with the previously reported immune response in NOD mice [22] and these data were used here to evaluate the possible immune regulatory activity of mycobacterial HSPs in co-cultures of APCs and T cells.

Hsp65 and Hsp70 proteins and DNAhsp65 have the ability to modulate co-cultured cells and change a Th1/Th17 to a Treg pattern of immune response. Although there was similar pattern of response between all immunomodulators, some small changes occurred that could be due to the own characteristics of the Hsp65 [7] and Hsp70 [8] used, as well as for the DNA vaccine construct [23]. The DNA construct must be internalized to be transcribed and release the Hsp65 protein endogenously [24]. Endogenous Hsp65 can act at different signaling pathways on APCs and T cells in relation to presented by the exogenous added protein [7]. Hsp65 and Hsp70, when extracellular, are recognized by cellular immune receptors [7, 8, 25]. The surprising discovery of HSP receptors on APCs afforded a molecular description of several immunological mechanisms [7]. Such recognition allows HSPs to be critical mediators of immune responses, initiating immune regulatory responses against cancers or pathogen-infected cells, exacerbating pre-existing inflammatory conditions, or suppressing ongoing immunity [7].

Hsp65 and Hsp70 can activate rodents and human macrophages and dendritic cells in vitro [8, 23, 26] and these early innate responses in turn can be funneled into and direct the type of adaptive immune response [7]. HSP-stimulated APCs also up-regulate CD80, CD86, CD40, and MHC class II [7]. We described here an up-regulation of IL −10 and suppression of IL-17, IL-6 and IFN-γ by immunomodulators in co-cultures. Note that the immuneregulatory products induced suppression in the secretion of IL- 17, both in the presence or absence of the specific antigen insulin. Moreover there was an up-regulation of expression of CD11b^+^CD86^+^ by APCs and CD4^+^CD25^hi^ by T cells. Moreover, there was an increase of T CD4^+^CD3^+^ across the stimuli used. This increase may be due to an increase of CD4^+^ cells producing IL-10 (Tr1 cells), and this is a point to be investigated. On the other hand, not necessarily only CD4^+^ cells could be CD25^hi^ secreting IL-10. Although not used antibodies specific for CD4^+^CD25^+^Foxp3^+^ that best characterizes the Treg cells by FACScan analyses, the use of CD4^+^CD25^hi^ markers of T cells in this work also phenotypically characterizes Treg cells [27–31].

The Th17 lineage of T helper cells can cause severe inflammatory diseases and needs to be regulated for their appropriate control of immune response [32]. It was recently demonstrated that Th17 cells transdifferentiate into regulatory cells by a change in their signature transcriptional profile and the acquisition of potent regulatory capacity contributing to the resolution of inflammation [32]. In recent years, much evidence has also been accumulated demonstrating that Treg cells can suppress auto aggressive T cell responses to host antigen, both in vitro and in vivo [27–31]. Treg cells were first described in autoimmune processes where the absence of CD25^+^ cells was associated with the development of autoimmune disease. Mechanisms by which Treg cells suppress auto aggressive responses are not completely known. Although cell–cell contact and the production of anti-inflammatory cytokines, such as IL-10 and TGF-β, have been implicated [33–35]. Our results are in line with this hypothesis. Some indications of the protection afforded by HSPs in various models of experimentally induced autoimmune diseases include the generation of Treg cells [7, 8] and the induction of anergy in auto reactive T cells. Thus, we propose that the presence of CD4^+^CD25^hi^ cells, together with the increased levels of IL-10 in co-cultures of NOD cells, indicate that Treg are up-regulate in this experimental model. Although not fully shown in this work, the results suggest that concomitant with the appearance of Treg cells, there was a significant suppression of Th17 lymphocytes, evaluated by the decrease of IL −17 and IL −6 cytokines in co-cultures. Evaluations at other time points of co-cultures with longer follow-up as well as in NOD animals treated with the immuneregulatory could be valuable to further investigate the potentially beneficial effects of HSPs in the immunotherapy of diabetes. These findings provide import clues to the design of new strategies for using HSPs in human immunotherapy.

## Conclusion

The in vitro model of up-regulation of cells from the immune system described here, showed for the first time that Hsp65 and Hsp70 proteins and DNAhsp65 have the ability to modulate co-cultured cells and change a Th1/Th17 to a Treg pattern of immune response. Thus, this study shows that immune cells from animals with an autoimmune disease can be imunoregulated in vitro in the presence of immunomodulators and open new perspectives for using this strategy in the research and development of new biopharmaceuticals for controlling diabetes and others autoimmune diseases, since this model is safe, effective, fast and easy to develop.

### Ethics approval

The experimental protocol was conducted according to the Ethical Principles In Animal Research adopted by the Brazilian College of Animal Experimentation (COBEA) and was approved by the Ribeirão Preto School of Medicine of University of São Paulo – Ethical Commission of Ethics in Animal Research (CETEA) (protocol 042/2011).

### Availability of supporting data

The data set (s) supporting the results of this article is (are) included within the article.

### Consent for publication

Not applicable.
